# Tryptophan–Kynurenine Pathway Activation and Cognition in Virally Suppressed Women With HIV

**DOI:** 10.1097/QAI.0000000000003454

**Published:** 2024-07-09

**Authors:** Eran Frank Shorer, Raha M. Dastgheyb, Audrey L. French, Elizabeth Daubert, Ralph Morack, Tsion Yohannes, Clary Clish, Deborah Gustafson, Anjali Sharma, Andre Rogando, Qibin Qi, Helen Burgess, Leah H. Rubin, Kathleen M. Weber

**Affiliations:** a Department of Neurology, Johns Hopkins Hospital, Baltimore, MD;; b Department of Medicine, Stroger Hospital of Cook County, Chicago IL;; c Hektoen Institute of Medicine, Chicago, IL;; d Metabolomics Platform, Broad Institute of Massachusetts Institute of Technology and Harvard, Cambridge, MA;; e Department of Neurology, State University of New York Downstate Medical Center, Brooklyn, NY;; f Department of Medicine, Albert Einstein College of Medicine, Bronx, NY;; g College of Science and Health, Charles R. Drew University of Medicine and Science, Los Angeles, CA;; h Department of Epidemiology and Population Health, Albert Einstein College of Medicine, Bronx, NY;; i Department of Psychiatry, University of Michigan, Ann Arbor, MI; and; Departments of j Psychiatry and Behavioral Sciences;; k Molecular and Comparative Pathobiology; and; l Epidemiology, Johns Hopkins University School of Medicine, Baltimore, MD.

**Keywords:** HIV, cognition, neuroinflammation, IDO, indoleamine 2,3-dioxygenase, TK pathway

## Abstract

**Background:**

Immune and cognitive dysfunction persists even in virally suppressed women with HIV (VS-WWH). Since inflammation and HIV proteins induce the enzyme indoleamine 2,3-dioxygenase (IDO), converting tryptophan (T) to kynurenine (K) while producing downstream neurotoxic metabolites, we investigated IDO activation (KT ratio) in relation to cognition in VS-WWH and demographically similar women without HIV (WWoH).

**Methods:**

Ninety-nine VS-WWH on stable antiretroviral therapy and 102 WWoH (median age 52 vs 54 years; 73% vs 74% Black, respectively) from the New York and Chicago sites of the Women's Interagency HIV Study (WIHS) completed a neuropsychological test battery assessing motor function, processing speed, attention/working memory, verbal fluency, verbal learning and memory, and executive function and had plasma measured for tryptophan–kynurenine metabolites through liquid chromatography-tandem mass spectrometry and monocyte-derived [soluble cluster of differentiation-14 (sCD14), soluble cluster of differentiation-163 (sCD163), monocyte chemoattractant protein-1 (MCP-1)] plus general inflammatory markers [tumor necrosis factor alpha-2 receptor (TNF-R2), high-sensitivity C-reactive protein, high-sensitivity interleukin-6] through enzyme-linked immunosorbent assays between 2017 and 2020.

**Results:**

VS-WWH had a higher KT ratio (*P* < 0.01) and higher sCD14 levels (*P* < 0.05) compared with WWoH. Higher sCD163 was associated with higher KT ratio (R = 0.29, *P* < 0.01) and worse fine motor function in VS-WWH; after adjusting for sCD163 and sCD14 in multivariable regressions, higher KT ratio remained significantly associated with impaired fine motor function in VS-WWH only (standardized β = −0.29, *P* < 0.05). IDO activation was not associated with cognition in WWoH.

**Conclusions:**

IDO activation (K:T) was associated with worse fine motor control in VS-WWH independent of measured systemic inflammation. Further studies investigating biological mechanisms linking IDO activation to fine motor function among VS-WWH are warranted.

## Abstract 2

Supplemental Digital Content is Available in the Text.

## INTRODUCTION

HIV is known to affect specific aspects of cognition; however, most studies have been performed in men and have studied cognition globally without considering individual domains.^1^ In women with HIV (WWH), the cognitive domains of learning and memory, processing speed, and motor function appear to be most vulnerable to the effects of HIV.^2^ Even in WWH who are virally suppressed WWH (VS-WWH), these cognitive complications persist or worsen over time, resulting in reduced quality of life and higher mortality.^3–8^ The mechanisms leading to cognitive dysfunction in VS-WWH are not fully known and warrant further investigation.

Although HIV does not directly infect neurons, HIV-related neural injury may occur due to ongoing neuroinflammation.^9,10^ HIV reservoirs within infected microglia and macrophages persist in the central nervous system by evading the immune system, thereby contributing to a chronic inflammatory state.^11,12^ Within this inflammatory milieu, the HIV transactivator of transcription protein and interferon-gamma induce the enzyme indoleamine-2,3-dioxygenase (IDO), which promotes the conversion of tryptophan (T) to kynurenine (K).^13–15^ This process may be a crucial component in the pathogenesis of cognitive dysfunction in PWH as IDO activation produces reactive oxygen species and the metabolite quinolinic acid (QA) from kynurenine, an N*-*methyl-D-aspartate receptor agonist that contributes to excitatory neurotoxicity.^16–19^

Higher levels of QA are related to impaired cognition, while the flux of tryptophan through IDO depletes serotonin and may contribute to HIV-associated neuropsychiatric manifestations, such as depression and sleep disturbances.^13,16,20–24^ Apart from damaging neurons, reactive oxygen species and QA further perpetuate the inflammatory response leading to a vicious cycle of neuroinflammation and neural injury. Plasma markers of inflammation and monocyte activation have been linked to cognitive outcomes; however, how this relates to plasma tryptophan and kynurenine is unexplored.^25–29^

The tryptophan–kynurenine (TK) pathway has been studied in the context of HIV. A recent review found increased IDO activation (using K:T ratio as a surrogate marker) is associated with age and chronic inflammation in PWH.^30^ Moreover, in the IDOze study, increased IDO activation (higher K:T ratios) in VS-WWH was associated with worse objective and subjective sleep outcomes.^22,23^ Tryptophan dysmetabolism and increased IDO activation have also been linked to depression and neurodegenerative disorders.^16,24,31,32^

Tryptophan metabolism has been studied in relation to global cognitive impairment in HIV; however, it remains unknown whether the TK pathway and IDO activation are implicated in *domain-specific* cognitive complications in VS-WWH. To date, the TK pathway and IDO activation have only been reported as associated with global cognitive deficits in a sample of predominately men with HIV.^20,32^ Here, we examine the association between TK pathway metabolites and domain-specific cognitive dysfunction in VS-WWH and demographically similar women without HIV (WWoH) coenrolled in the Women's Interagency HIV Study (WIHS) and “IDOze” substudy of sleep and the TK pathway in WWH and WWoH.^22,23^

We hypothesized that greater IDO activation will be present in the VS-WWH and that IDO activation will be correlated with domain-specific impairments in cognition in both VS-WWH and WWoH.

## METHOD

### Participants

We analyzed TK pathway metabolites from women coenrolled in the WIHS and IDOze Study between October 2018 and January 2020^23^ at the Chicago and New York City sites. The WIHS is a longitudinal study of WWH and demographically similar WWoH and has been previously described in detail.^33,34^ IDOze is a substudy partially nested within the WIHS examining the TK pathway in relation to sleep and circadian dysfunction.^28^ WIHS women were eligible to coenroll in IDOze if English-speaking and aged 35–70 years; WWH were on stable antiretroviral therapy (ART) excluding efavirenz, with an HIV RNA level <200 copies/mL and CD4^+^ T Lymphocyte count ≥200 cells/µL in the 6 months before enrollment. Exclusion criteria included severe chronic or acute psychiatric and/or medical illness including uncontrolled hypertension or diabetes, narcolepsy, illicit drug use (>1 d/wk of self-reported use), use of psychotropic medication and/or sleep aids (prescription hypnotics, over the counter sleeping aids >2 nights per week, melatonin supplementation), night-shift work, pregnancy or lactation within past 3 months, use of estrogen-containing contraceptives, or hormone replacement therapy. Written informed consent was obtained from all participants in accordance with Department of Health and Human Services guidelines and the institutional review boards at each participating site.

### Laboratory Methods

#### Plasma Tryptophan and Kynurenine

Blood samples were processed within 24 hours of collection; plasma aliquots were stored at −70°C until batch testing. We extracted total (bound and unbound) metabolites of interest from plasma using liquid chromatography–tandem mass spectrometry, reporting peak intensity for each metabolite (Broad Institute Metabolomics Platform of MIT/Harvard). Specifically, a 6495 triple quadrupole mass spectrometer coupled to a 1290 Infinity II U-HPLC system (Agilent, Santa Clara, CA) was used to quantify plasma tryptophan and kynurenine. Raw data were analyzed using MassHunter software (Agilent) for automated peak integration, and the quality of integration was manually reviewed and compared against reference standards to confirm the identities of the metabolites. The coefficients of variation (CV) for tryptophan and kynurenine are 4.2% and 3.37%, respectively.

#### Plasma Inflammatory Markers

We assayed selected general inflammatory markers [high-sensitivity interleukin (hsIL)-6, high-sensitivity C-reactive protein (CRP), and tumor necrosis factor alpha-2 receptor (TNFR2)], and markers that reflect monocyte activation and neuroinflammation in the context of HIV [soluble cluster of differentiation-14 (sCD14), soluble cluster of differentiation-163 (sCD163), and monocyte chemoattractant protein (MCP)-1]. Sandwich enzyme-linked immunosorbent assays (ELISAs) were run in duplicate with means used to quantify inflammatory markers: MCP-1, sCD14, sCD163, TNFR2; hsIL-6 levels were quantified using Quantikine ELISA kits (R&D Systems, Minneapolis, MN), and high-sensitivity CRP levels were quantified using human high-sensitivity ELISA kits (Elabscience; Houston, TX). Absorbances were measured using the BioTek ELx800 plate reader and analyzed using the Gen5 2.0 software. The Gen5 2.0 software calculated a standard curve based on internal controls and calibrators and a quantitative result for unknown samples. A CV cutoff of 10% was used to determine replicate precision for all ELISA assays; if the replicates had a CV below this value, the mean of the replicates was used in analyses. Any replicates with a CV >10% were retested. Fewer than 1% of samples needed to be retested.

#### Cognition

Neuropsychological (NP) testing was performed within 3 years of the plasma tryptophan and kynurenine samples (median = 693 days, interquartile range = 361 days). The NP test battery assessed the following cognitive domains: motor function, processing speed, attention/working memory, verbal fluency, verbal learning and memory, and executive function. Motor function was assessed with Grooved Pegboard (outcomes = time to complete dominant and nondominant hand); processing speed with Symbol Digit Modalities Test (outcome = total correct) and Stroop trial 2 (outcome = time to complete); attention/working memory with the Letter–Number Sequencing (outcomes = total correct on the control/attention condition and working memory condition); verbal fluency with letter and animal fluency (outcomes = total correct); verbal learning and memory (Hopkins Verbal Learning Test; outcomes = total learning across trials and delay free recall); and executive function with Trail Making Test-part B and Stroop-interference trial (outcomes = time to completion). All timed outcome measures were log transformed and multiplied by −1 so higher values equated to better performance. Demographically adjusted T-scores were derived for each outcome (adjusted for age, reading ability through Wide Range Achievement Test-Revised, education, race/ethnicity); see Rubin et al^3^ and Maki et al^35,36^ for details. T-scores were used to create domain scores.

#### Participant Demographic and Clinical Data

Demographic information, including age, race/ethnicity, self-reported menopausal status, and recent substance use (alcohol, cigarettes, marijuana, and illicit drugs in the past 6 months), and presence of depressive symptoms were extracted from the most recent WIHS study visit or newly collected during the IDOze visit via sleep diaries. Participants' height and weight were measured and used to calculate body mass index (BMI, in kg/m^2^). In this analysis, we defined HIV viral suppression as undetectable (HIV RNA level < 20 copies/mL per kit lower limit of detection) and excluded women with viremia. Depressive symptoms, which can confound assessments, were measured using the Center for Epidemiological Studies Depression Scale.

#### Statistical Methods

VS-WWH were compared with WWoH using χ^2^ measures of association for categorical variables, Wilcoxon rank-sum to compare means of nonparametric continuous variables, and one-way analysis of variances to compare parametric continuous variables. Owing to skewed distributions, all plasma metabolites and inflammatory markers were winsorized to 3 SDs from the mean and log transformed. The KT ratio was created after log transforming. Multivariable linear regressions models were utilized to identify relationships between kynurenine, tryptophan, and the KT ratio and domain-specific cognitive function while controlling for age, the number of days between blood draw and NP testing, self-reported fasting status, BMI, and smoking status. All analyses were conducted using R software (version 4.2.2; R Foundation for Statistical Computing) and significance was set at *P* < 0.05. The Benjamini and Hochberg procedure was used to control the false discovery rate (FDR) across the series of regression analyses.

## RESULTS

Table 1 provides sociodemographic and clinical characteristics for the 99 VS-WWH and 102 WWoH. The VS-WWH had a median age of 54, and 73% were Black. The WWoH had a median age of 52, with 74% being Black. Overall, the 2 groups were similar across most factors, although WWoH were more likely to smoke tobacco compared to VS-WWH (*P* < 0.01). There were no associations between the KT ratio, tryptophan, or kynurenine with respect to Center for Epidemiological Studies Depression Scale scores, CD4 count, CD4 nadir, and duration of years on ART. There was a significant correlation between age and the KT ratio in the whole sample (R = 0.31, *P* <0.001).

**TABLE 1.** Participant Demographics Comparing VS-WWH With WWoH

Variable	N	WWoH, N = 102	VS-WWH, N = 99	*P* *
Age, median (IQR)	201	52 (46–58)	54 (47–59)	0.15
Race/ethnicity, n (%)	201			0.07
Non-Hispanic Black		75 (74)	72 (73)	
Hispanic		27 (26)	21 (21)	
Other		0 (0)	3 (3)	
Non-Hispanic White		0 (0)	3 (3)	
Highest level of education, n (%)	120			0.68
Above high school		29 (49)	26 (43)	
Below high school		1 (1.7)	2 (3.3)	
High school		29 (49)	33 (54)	
Postmenopausal, n (%)	165	46 (53)	48 (61)	0.35
BMI, median (IQR)	200	31 (27–35)	31 (27–37)	0.68
Prevalent diabetes (controlled), n (%)	201	27 (26)	28 (28)	0.77
CES-D (depression) score, median (IQR)	201	5 (3–11)	6 (3–15)	0.14
Fasted at metabolite blood draw, n (%)	199	90 (90)	89 (90)	0.98
Current smoker, n (%)	201	47 (46)	27 (27)	**0.006**
Current alcohol use, n (%)	196			0.11
Abstainer		49 (50)	58 (59)	
>0–7 drinks/week		41 (42)	38 (39)	
>12 drinks/week		8 (8)	2 (2)	
Recent illicit drug use, n (%)	199	7 (7)	2 (2)	0.17
CD4 count, median (IQR)	97		747 (542–979)	
Nadir CD4 count, median (IQR)	99		222 (108–360)	

All *p* values that are statistically significant (*p* < 0.05) are bolded.

* Wilcoxon rank sum test; Fisher exact test; Pearson χ^2^ test.

CES-D, Center for Epidemiological Studies Depression Scale; IQR, interquartile range; recent use, past 6 months.

VS-WWH had a significantly higher KT ratio compared to WWoH (*P* < 0.01), due to higher levels of kynurenine (Table 2). VS-WWH also exhibited higher levels of sCD14 compared to WWoH (*P* = 0.05). There were no significant differences by HIV status in plasma tryptophan, sCD163, MCP-1, or inflammatory cytokines (TNFR2, IL-6, CRP). There were no statistically significant differences in cognitive performance domains between VS-WWH and WWoH (see Table 1, Supplemental Digital Content, http://links.lww.com/QAI/C299), though there was a trend toward higher performance among WWoH in several domains.

**TABLE 2.** Biochemical Differences Between VS-WWH and WWoH

Biochemical, Median (IQR)	N	WWoH, N = 102	VS-WWH, N = 99	*P* *
KT ratio	201	0.654 (0.642–0.669)	0.662 (0.649–0.684)	**0.005**
Kynurenine (peak intensity)	201	8.90 (8.62–9.18)	9.01 (8.75–9.26)	**0.03**
Tryptophan (peak intensity)	201	13.62 (13.42–13.77)	13.59 (13.39–13.76)	0.60
sCD14, pg/mL	201	13.95 (13.81–14.19)	14.04 (13.92–14.20)	**0.03**
sCD163, ng/mL	201	5.90 (5.57–6.25)	5.98 (5.62–6.34)	0.22
MCP-1, pg/mL	201	5.18 (4.92–5.49)	5.28 (4.94–5.56)	0.36
TNFR2, pg/mL	201	7.51 (7.27–7.75)	7.61 (7.39–7.83)	0.06
hsIL6, pg/mL	201	0.61 (0.23–1.35)	0.82 (0.35–1.27)	0.41
hsCRP, ng/mL	201	7.85 (6.98–8.73)	7.98 (7.12–8.64)	0.87

All *p* values that are statistically significant (*p* < 0.05) are bolded.

All data were log transformed and winsorized to 3 SDs to account for outliers.

* Wilcoxon rank sum test.

IL-6, high sensitivity interleukin-6; IQR, interquartile range; KR ratio, kynurenine–tryptophan ratio.

### Associations Between the KT Pathway and Cognition

**FIGURE 1.**
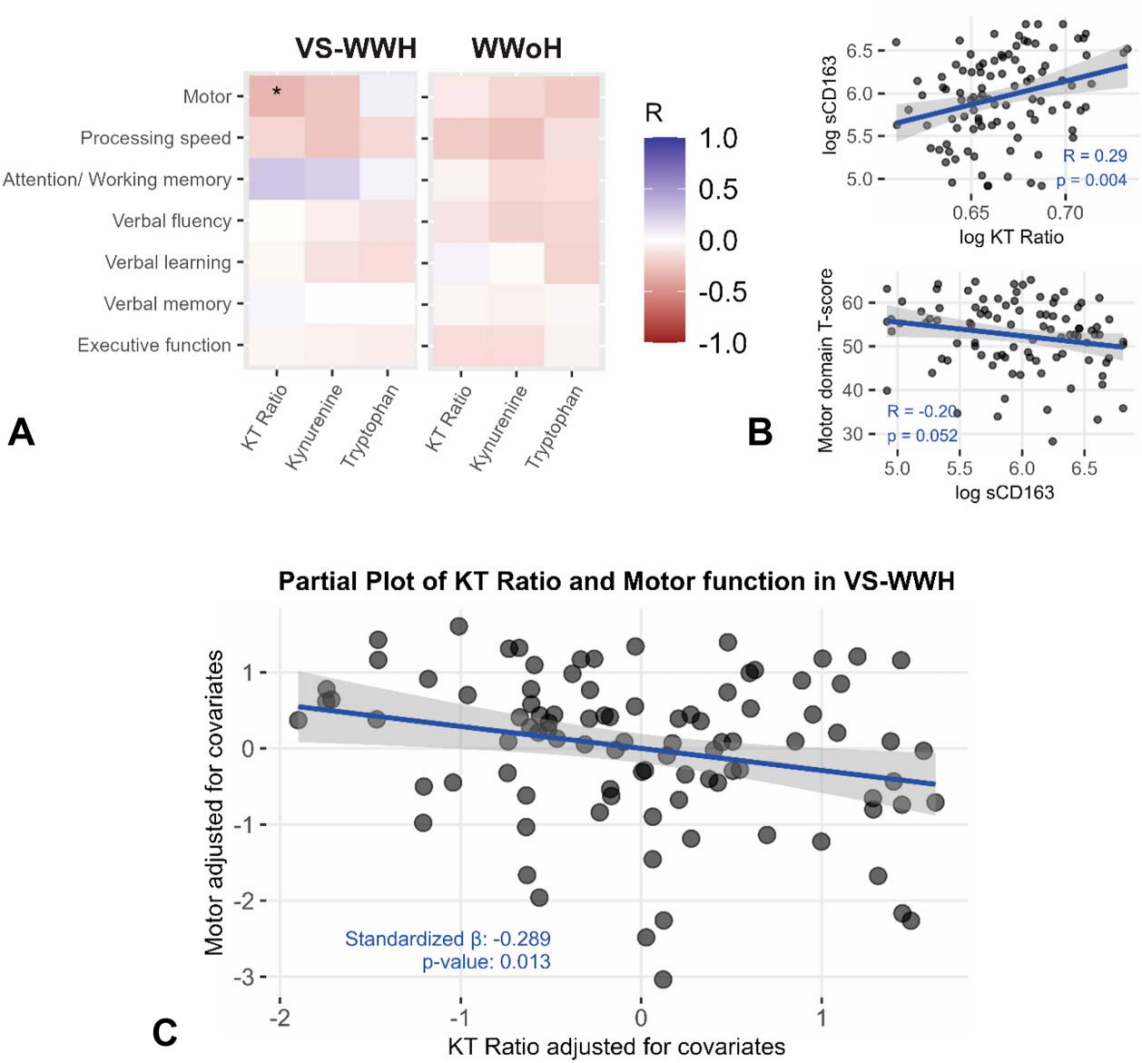
Kynurenine–tryptophan (KT) ratio predicts motor impairment in VS-WWH but not in WWoH. A, Higher KT ratio is associated with worse complex motor domain T-scores in VS-WWH. Heatmaps show the partial correlation coefficient for relationships between KT ratio, kynurenine, and tryptophan across cognitive domains in VS-WWH and WWoH. Spearman correlation coefficient was used to determine partial correlations controlling for age, days between neuropsychological testing and metabolite draw, fasting status at metabolite draw, smoking status, BMI, and sCD14. Benjamini and Hochberg procedure was used for FDR correction; *P < 0.05; (B) higher log sCD163 is associated with an increased log KT ratio and poorer motor function (C) partial plot for the KT ratio and motor domain in VS-WWH after normalizing all variables. The same covariates, with the inclusion of sCD163, were used as in (A). Greater IDO activation (higher KT ratio) predicts worse motor performance independent of sCD163.

Figure 1A depicts the FDR-corrected partial correlations between each cognitive function and kynurenine, tryptophan, and the K:T. In VS-WWH but not WWoH, higher K:T was significantly associated with decreased motor function assessed via time to complete the Grooved Pegboard Test (*P* < 0.05). A nominal significant association was observed between the K:T and Attention and Working Memory in VS-WWH only (raw *P* < 0.05, R = 0.26), additional non-FDR corrected associations are shown in Table 2, Supplemental Digital Content http://links.lww.com/QAI/C300. In a multivariable regression model, we included age, the number of days between NP testing and blood draw, sample fasting status, smoking status, BMI, and sCD14. In this model only the inverse association between the K:T and motor function [standardized beta (β) = −0.33, *P* < 0.01] remained significant among VS-WWH.

We found that higher sCD163, a biomarker linked to low grade inflammation and macrophage activation, was correlated with higher K:T (*R* = 0.29, *P* < 0.01) and worse motor function in VS-WWH (*R* = −0.2, *P* = 0.05; Fig. 1B). To determine if the relationship between K:T and motor function among VS-WWH was accounted for by inflammation, we reran the multivariable linear regression (Fig. 1C) with the addition of sCD163. In this model, K:T, but not sCD163, was significantly associated with motor function (standardized β = −0.29, *P* < 0.05).

## DISCUSSION

We investigated the relationship between IDO activation (K:T) and domain-specific cognitive function in the context of HIV viral suppression in women. Our findings indicate that in VS-WWH, but not in WWoH, a higher K:T and higher sCD163 (monocyte activation and low-grade inflammation marker) were both associated with worse motor function (grooved pegboard performance). Another inflammatory marker, sCD14, was significantly higher in VS-WWH relative to WWoH. When K:T, sCD163, and sCD14 were entered into models predicting motor function only K:T remained significant in VS-WWH, suggesting IDO activation is associated with impaired motor function independent of these inflammatory markers.

In our investigation we observed no significant cognitive impairment or serostatus differences across cognitive domains. However, it is crucial to interpret these findings within the constraints of our sample size which was likely underpowered to detect such differences. This caveat is important when juxtaposing our results against the well documented decline in motor function and other cognitive faculties among PWH that is evident in extensive longitudinal studies such as the Multicenter AIDS Cohort Study,^37^ the WIHS,^3^ and the CNS HIV Anti-Retroviral Therapy Effects Research study.^38^

Motor function in this study was measured using the Grooved Pegboard. As the Grooved Pegboard is a highly integrated task examining multiple motor functions including manual dexterity, fine motor control, visual motor coordination, and speed, it is difficult to distinguish which aspect (or aspects) is truly impaired. We measured the Grooved Pegboard task using time to completion; however, evaluating number of peg drops may be another useful indicator of complex motor performance. Although the Grooved Pegboard gives an overall impression of complex motor performance, it may not directly translate to performance of activities of daily living. More sensitive and specific tests are needed to evaluate the functional impact of motor decline in PWH.^39,40^ Dysfunction in other components of motor function such as muscle strength, coordination, and gait (which were not evaluated in this analysis) have also been observed in the context of HIV^41–43^; future studies are required to identify whether IDO activation is associated with deficits across the spectrum of motor functioning in HIV.

IDO activation is strongly associated to aging and chronic inflammatory conditions such as osteoporosis, and independently predicts mortality in PWH on ART.^30,44^ In our study, age was significantly associated with the KT ratio, and as such was used as a covariate in our models. Although we also included relevant inflammatory markers such as CD163 and CD14 as covariates in our model, we could not account for the full extent of inflammation. Furthermore, we did not exclude participants with chronic inflammatory conditions such as osteoporosis that are known to induce IDO.

Persistent neuroinflammation may underlie motor dysfunction in HIV. The presence of pathological lesions in regions responsible for motor function is associated with the extent of monocyte and microglial infiltration in those areas.^45–47^ Moreover, markers of monocyte activation such as sCD163, sCD14, and MCP-1 correlate with cognitive and motor dysfunction, and remain elevated despite viral suppression.^2,25–27,48–51^ We found that higher sCD163 was associated with worse motor function; however, sCD163 was not significant in models that included K:T.

Our findings differ from Rubin et al,^52^ which showed that immune activation was associated with motor dysfunction in both VS-WWH and WWoH, perhaps due to our limited sample size. Immune activation can be driven by chronic inflammatory conditions other than HIV. Differing prevalence of chronic inflammatory conditions might explain the lack of association between motor dysfunction and inflammatory markers in our group of WWoH. Endothelial damage and vascular dysfunction can also contribute to cognitive deficits in both in VS-WWH and WWoH^52,53^ and should be examined in future studies. Although we controlled for BMI and smoking status in our analyses, we did not include measures of endothelial dysfunction which may differ between groups.

Although we showed an association between IDO activity and monocyte activation, IDO influences other aspects of immune regulation, notably the suppression of T-Cell activation^54^ in chronic inflammatory states. IDO activation has been shown as a critical inciting event leading to a reduced number of Th_17_ cells (sentinel immune cells in the gut lining), ultimately leading to increased gastrointestinal bacterial translocation and a chronic inflammatory state.^55^ IDO activation can both indirectly cause neural damage due to sustained inflammation and directly generate neurotoxic kynurenine pathway by-products such as QA.^20^

Another possible factor resulting in cognitive dysfunction in HIV is a hyper-reactive antiviral response involving monocyte-derived interferons.^56,57^ Interferons are known inducers of IDO^58^ and may be an important mediator linking HIV infection, IDO activation, and motor dysfunction. Genetic differences in immune responses to HIV and differential HIV-1 expression in particular brain regions may explain why not all VS-WWH have increased IDO activation or exhibit motor dysfunction.^59^

Our study acknowledges several limitations. Our current cross-sectional approach limited our ability to infer any causative effects of IDO activation on cognition in VS-WWH. Our method of mass spectrometry produced only relative quantities of each metabolite, rather than absolute concentration of metabolites, thus limiting our ability to compare values across studies and across populations. The average interval between the NP test battery and the metabolite blood draw was approximately 1 year and 10 months (given that cognition is tested every 2 years in this cohort). Concurrent cognitive testing and blood draw may have revealed more pronounced associations between motor function and the K:T, especially given the decline in motor function over time. Despite the observed stability of the K:T ratio over time in contexts other than HIV, as supported by several studies,^60–62^ our investigation will continue to monitor the K:T ratio's stability through subsequent metabolomic analyses within this cohort. Although we excluded male participants, existing literature suggests an absence of sex differences in the K:T ratio but a heightened sensitivity to serotonergic alterations in female participants.^63^ To examine sex-difference, our follow-up study will incorporate a male cohort. Finally, we did not account for diet, and gut microbiota which are determinants of plasma tryptophan concentration,^64^ nor did we account for specific ART regimens, which may have affected our results given newer integrase-inhibitors based regimens are associated with better cognitive function.^65^

This study is the first to investigate IDO activation in relation to cognition regarding viral suppression in women with HIV and possesses distinctive strengths. Instead of studying cognition globally, we studied domain-specific cognition. Cognitive domains are differentially affected in HIV, and the pathogenetic mechanisms contributing to dysfunction across these domains may also differ.^3,52^ Studying these domains separately allowed us to identify that performance in the motor domain is associated with changes in tryptophan metabolism in our sample of VS-WWH.

Another strength of our study was that we excluded women with acute medical conditions (uncontrolled hypertension or diabetes, recent infection, or cancer) and severe psychiatric illness or who were on psychotropic medication, women who reported illicit substance use (>1 d/wk), and women who were unable to refrain from heavy alcohol use, all factors that could both impair performance on the Grooved Pegboard Test and impact TK pathway metabolites. In a sample inclusive of this population, we might expect an even more profound association between IDO activation and impaired motor function. Another strength of this study was the incorporation of plasma inflammatory markers, allowing us to show that the association between IDO activation and motor function in VS-WWH was persistent even after controlling sCD14 and sCD163.

In conclusion we found that IDO activation was associated with poorer fine motor skills in midlife VS-WWH but not in demographically similar WWoH. Larger longitudinal studies are required to investigate the causal relationships linking HIV infection, neuroinflammation, and IDO activation to motor dysfunction and the implications of these findings on chronic disease management and other activities of daily living among women as they age with HIV. Future studies could also consider investigating the use of L-tryptophan supplementation for those with high IDO activity.

## Supplementary Material
