# Supplementary material for: Tryptophan–Kynurenine Pathway Activation and Cognition in Virally Suppressed Women With HIV
Source: J Acquir Immune Defic Syndr. 2024 Jul 9;96(5):494–500. doi: 10.1097/QAI.0000000000003454 (PMC11236271; doi:10.1097/QAI.0000000000003454)
Supplement: Supplementary file 1 [file qai-96-494-s001.docx]

## Supplementary Table 1:

| Cognitive Domain T-Score, median (IQR) | N | WWoH,  N = 102 | VS-WWH,  N = 99 | P-value^1^ |
| --- | --- | --- | --- | --- |
| **Motor** | 196 | 52 (47 – 58) | 54 (47 – 58) | 0.47 |
| **Processing speed** | 199 | 50 (43 – 57) | 51 (46 – 55) | 0.68 |
| **Attention/working memory** | 192 | 49 (43 – 55) | 49 (43 – 56) | 0.98 |
| **Verbal fluency** | 199 | 51 (45 – 57) | 52 (47 – 57) | 0.21 |
| **Verbal learning** | 199 | 49 (41 – 56) | 50 (41 – 55) | 0.90 |
| **Verbal memory** | 199 | 49 (42 – 57) | 52 (43 – 60) | 0.09 |
| **Executive function** | 199 | 50 (44 – 57) | 53 (45 – 58) | 0.08 |
| **Supplementary Table 1: Cognitive performance in virally suppressed women with HIV (VS-WWH) and women without HIV (WWoH).** ^1^ Wilcoxon rank sum test; HIV = human immunodeficiency virus; IQR = interquartile range | | | | |
