# Supplementary material for: Tryptophan–Kynurenine Pathway Activation and Cognition in Virally Suppressed Women With HIV
Source: J Acquir Immune Defic Syndr. 2024 Jul 9;96(5):494–500. doi: 10.1097/QAI.0000000000003454 (PMC11236271; doi:10.1097/QAI.0000000000003454)
Supplement: Supplementary file 2 [file qai-96-494-s002.docx]

## Supplementary Table 2:

| \|  \| **VS-WWH** \| \| \| \| \| \|  \| **WWoH** \| \| \| \| \| \| \| --- \| --- \| --- \| --- \| --- \| --- \| --- \| --- \| --- \| --- \| --- \| --- \| --- \| --- \| \|  \| **KT Ratio** \| \| **Kynurenine** \| \| **Tryptophan** \| \|  \| **KT Ratio** \| \| **Kynurenine** \| \| **Tryptophan** \| \| \|  \| **r** \| **p** \| **r** \| **p** \| **r** \| **p** \|  \| **r** \| **p** \| **r** \| **p** \| **r** \| **p** \| \| **Motor** \| -0.333 \| **0.001** \| -0.259 \| **0.014** \| 0.069 \| 0.520 \|  \| -0.100 \| 0.334 \| -0.177 \| 0.087 \| -0.233 \| **0.023** \| \| **Processing Speed** \| -0.182 \| 0.080 \| -0.257 \| **0.013** \| -0.169 \| 0.106 \|  \| -0.230 \| **0.025** \| -0.280 \| **0.006** \| -0.140 \| 0.177 \| \| **Attention/Working memory** \| 0.263 \| **0.013** \| 0.221 \| **0.038** \| 0.061 \| 0.571 \|  \| -0.052 \| 0.619 \| -0.163 \| 0.120 \| -0.150 \| 0.152 \| \| **Verbal fluency** \| -0.011 \| 0.918 \| -0.073 \| 0.485 \| -0.123 \| 0.241 \|  \| -0.116 \| 0.262 \| -0.201 \| 0.051 \| -0.178 \| 0.085 \| \| **Verbal learning** \| -0.032 \| 0.764 \| -0.132 \| 0.208 \| -0.160 \| 0.125 \|  \| 0.060 \| 0.567 \| -0.021 \| 0.836 \| -0.194 \| 0.059 \| \| **Verbal memory** \| 0.030 \| 0.773 \| 0.006 \| 0.956 \| 0.001 \| 0.995 \|  \| -0.044 \| 0.673 \| -0.063 \| 0.546 \| -0.038 \| 0.717 \| \| **Executive function** \| -0.040 \| 0.700 \| -0.062 \| 0.554 \| -0.078 \| 0.457 \|  \| -0.154 \| 0.136 \| -0.157 \| 0.129 \| -0.050 \| 0.632 \| |  |  |  |  |
| --- | --- | --- | --- | --- | --- | --- | --- | --- | --- | --- | --- | --- | --- | --- | --- | --- | --- | --- | --- | --- | --- | --- | --- | --- | --- | --- | --- | --- | --- | --- | --- | --- | --- | --- | --- | --- | --- | --- | --- | --- | --- | --- | --- | --- | --- | --- | --- | --- | --- | --- | --- | --- | --- | --- | --- | --- | --- | --- | --- | --- | --- | --- | --- | --- | --- | --- | --- | --- | --- | --- | --- | --- | --- | --- | --- | --- | --- | --- | --- | --- | --- | --- | --- | --- | --- | --- | --- | --- | --- | --- | --- | --- | --- | --- | --- | --- | --- | --- | --- | --- | --- | --- | --- | --- | --- | --- | --- | --- | --- | --- | --- | --- | --- | --- | --- | --- | --- | --- | --- | --- | --- | --- | --- | --- | --- | --- | --- | --- | --- | --- | --- | --- | --- | --- | --- | --- | --- | --- | --- | --- | --- | --- | --- | --- |
|  |  |  |  |  |
|  |  |  | |  |
| **Supplementary Table 2: Partial correlations between cognitive domains and tryptophan-kynurenine metabolites in virally suppressed women with HIV (VS-WWH) and women without HIV (WWH).** Spearman’s correlation coefficient (r), p-values determined after controlling for: smoking status, days apart between neuropsychological testing and metabolite draw, fasting status at metabolite draw, BMI, and sCD14. HIV = human immunodeficiency virus. |  |  | |  |
|  |  |  | |  |
